# ﻿*Didymocarpuspingyuanensis* (Gesneriaceae), a new species endemic to Danxia landscape from Guangdong Province, China, and two new combinations in *Didymocarpus*

**DOI:** 10.3897/phytokeys.244.126137

**Published:** 2024-07-16

**Authors:** Ling-Han Yang, Jing-Min Dai, Fang Wen, Jian-Hui Liu, Xue-Zheng Lan, Qiang Fan

**Affiliations:** 1 State Key Laboratory of Biocontrol and Guangdong Provincial Key Laboratory of Plant Stress Biology, School of Life Sciences, Sun Yat-sen University, Guangzhou 510275, China; 2 National Park and Nature Education Research Institute, Sun Yat-sen University, Guangzhou 510275, China; 3 Guangxi Institute of Botany, Guangxi Zhuang Autonomous Region and Chinese Academy of Sciences, Guilin 541006, Guangxi, China; 4 National Gesneriaceae Germplasm Resources Bank of GXIB, Gesneriad Committee of China Wild Plant Conservation Association, Gesneriad Conservation Center of China, Guilin Botanical Garden, Chinese Academy of Sciences, Guilin 541006, Guangxi, China; 5 Pingyuan County Wuzhishi Provincial Scenic Spot, Meizhou 514625, China; 6 Pingyuan County Forestry Bureau, Meizhou 514699, China

**Keywords:** *Didymocarpusheucherifolius* var. *gamosepalus*, *Didymocarpusheucherifolius* var. *yinzhengii*, flora of Danxia, taxonomy

## Abstract

*Didymocarpuspingyuanensis*, endemic to the Danxia landscape in Pingyuan County, Guangdong, China, is described and illustrated here. This species can be distinguished from other members of Didymocarpussect.Heteroboea by its calyx deeply 5-lobed to about three quarters of its length. The phylogenetic position of the new species within *Didymocarpus* was examined using nuclear ribosomal internal transcribed spacer (ITS) sequences. Based on phylogenetics analysis and morphological evidence, we propose two new combinations, elevating the two varieties to species level, namely *D.yinzhengii* and *D.gamosepalus*.

## ﻿Introduction

*Didymocarpus* Wall. was once a large genus with approximately 200 species (Weber and Brutt 1998). Recent molecular phylogenetic studies and morphological revisions of *Didymocarpus* have led to a reduction in the estimated number of species from 200 down to 60–80, with some species being transferred to *Henckelia* Spreng., *Hovanella* A.Weber & B.L.Burtt, *Petrocodon* Hance (Weber and Brutt 1998; Weber et al. 2000, 2011; Möller and Clark 2013).

For species of this genus in China, Wang et al. (1990) classified them into two sections: sect. Didymocarpus (herbs with stems) and sect. Heteroboea W.T.Wang auct. non Benth (herbs without stems). Sect. Heteroboea were initially defined by morphological characters, but on the basis of recent systematic results and morphological comparison, four species have been assigned to the genus *Petrocodon*, these species are *P.bonii* (Pellegr.) A.Weber & Mich.Möller, *P.mollifolius* (W.T.Wang) A.Weber & Mich.Möller, *P.niveolanosus* (D.Fang & W.T.Wang) A.Weber & Mich.Möller, *P.hancei* (Hemsl.) Mich.Möller & A.Weber (Weber et al. 2011). Recently, four new taxa within this section were discovered and published: *Didymocarpusdissectus* F.Wen, Y.L.Qiu, Jie Huang & Y.G.Wei (Wen et al. 2013) from Fujian Province, D.heucherifoliusHand.-Mazzvar.yinzhengii J.M.Li & S.J.Li (Li and Li 2014) from Hunan Province, D.heucherifoliusHand.-Mazzvar.gamosepalus Xin Hong & F.Wen (Xu et al. 2019) from Guangdong, China, and *D.lobulatus* F.Wen, Xin Hong & W.Y.Xie (Xie et al. 2020) from Zhejiang, China. In addition, *D.subpalmatinervis* W.T.Wang, which was placed in sect. Heteroboea has been transferred to *Petrocodon* as a new combination with *P.subpalmatinervis* (W.T.Wang) F.Wen & Z.L.Li after a thorough study (Li et al. 2023). Thus, before the completion of the revision work for this article, there were eight species and two varieties in sect. Heteroboea, all of which are endemic to China.

During a field investigation of Danxia landscapes in Pingyuan County, Guangdong, in April 2023, we encountered a *Didymocarpus* species in bloom and confirmed its classification within sect. Heteroboea of this genus as it was stemless herb (Wang et al. 1990). However, it differs from all known species in this section by having a calyx that is deeply 5-lobed to about three quarters of its length. After thorough morphological comparisons using herbarium specimens, digital images, and relevant literature on other similar species, we concluded that this plant represents an undescribed species. Here, the putative species is described and illustrated based on morphological observations and compared with closely related species. Additionally, we used nuclear DNA internal transcribed spacer (ITS) to reconstruct the phylogeny to evaluate the phylogenetic position of *Didymocarpuspingyuanensis*.

## ﻿Material and methods

### ﻿Morphological study

We used a micrometer and a stereomicroscope to observe and measure the morphological traits of the putative species. Morphological comparisons between *Didymocarpuspingyuanensis* and its related species were based on dry specimens we collected, relevant literature (Wen et al. 2013; Li and Li 2014; Xu et al. 2019; Xie et al. 2020), as well as digital images on the Chinese Virtual Herbarium (https://www.cvh.ac.cn/) and the China Field Herbarium (https://www.cfh.ac.cn/). Morphological observation was conducted in the Herbarium of Sun Yat-sen University (SYS).

### ﻿Taxon sampling and molecular analysis

The ITS region was used for examining the phylogenetic position of the putative species. During May 2023, we collected 6 taxa belonging to sect. Heteroboea for this study. These taxa included *Didymocarpuscortusifolius* (Hance) H.Lév., *D.salviiflorus* Chun, *D.lobulatus*, D.heucherifoliusvar.heucherifolius Hand.-Mazz, D.heucherifoliusvar.yinzhengii, D.heucherifoliusvar.gamosepalus. Except for D.heucherifoliusvar.gamosepalus, all species were collected from their type localities. The population of D.heucherifoliusvar.gamosepalus at its type locality has gone extinct due to human disturbance caused by nearby village activities. Consequently, we were unable to find it at its type locality in Pingyuan County, Guangdong. Instead, we collected specimens from Zijin County, Guangdong. Voucher specimens were deposited in SYS. We sequenced their ITS gene sequences to verify the molecular differences; the sequences have been uploaded to GenBank. Thirty-eight species of *Didymocarpus* and related genera with ITS gene sequences in NCBI GenBank were downloaded for analysis. In total, we got 45 ITS gene sequences of 45 taxa, GenBank accession numbers were followed after their Latin name in the phylogenetic tree. These taxa encompass three *Gyrocheilos* W.T.Wang species, one *Allocheilos* W.T.Wang species, one *Raphiocarpus* Chun species, six *Primulina* species, three *Petrocodon* species, nineteen species from Didymocarpussect.Didymocarpus and nine from Didymocarpussect.Heteroboea. *Sinningiaincarnata* (Aubl.) D.L.Denham and *S.tubiflora* Fritsch were selected as outgroups. All but one species of Didymocarpussect.Heteroboea were included in this study. The excluded species was *Didymocarpusreniformis* W.T.Wang, which was not located during multiple expeditions, and only imprecise locality data were available.

Total DNA was extracted from silica-gel-dried leaves using the modified cetyltrimethylammonium bromide (CTAB) protocol (Doyle and Doyle 1987). Polymerase chain reaction was carried out based on the program setting as proposed by Lee et al. (2022) using the universal primers, ITS1 and ITS4 (White et al. 1990). The ITS sequence was aligned with Clustal W which is embedded in MEGA-11 (Tamura et al. 2021). The sequences in the alignment were manually adjusted. Phylogenetic trees were reconstructed using the maximum likelihood (ML) methods and Bayesian inference (BI) methods via MEGA-11 (Tamura et al. 2021) and MrBayes v3.2.1 (Ronquist et al. 2012). The optimum DNA substitution model calculated using the “Find best DNA/Protein Models (ML) function embedded in MEGA-11 was Kimura 2-parameter model (K2) with Gamma Distributed With Invariant Sites (G+I) (=K2+G+I). All branch nodes were calculated with 1000 bootstrap (BS) replicates. BI analysis employed random starting trees and four Markov chain Monte Carlo (MCMC) simulations were run simultaneously and sampled every 1000 generations for 1 million generations. Bayesian posterior probabilities (PP) were calculated as the majority consensus of all sampled trees with the first 25% discarded as burn-in.

## ﻿Results and discussion

### ﻿Molecular analysis

**Figure 1.**
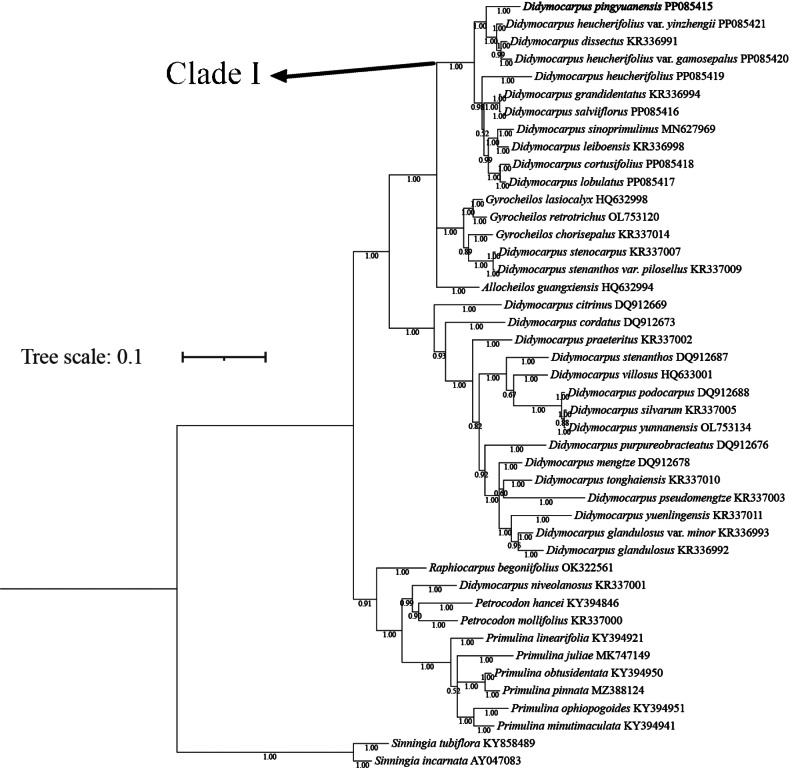
Bayesian inference (BI) tree based on ITS sequences of the new species *Didymocarpuspingyuanensis* and related species. Bayesian posterior probabilities are shown along the branches. The new species described in this study is shown in bold.

**Figure A1.**
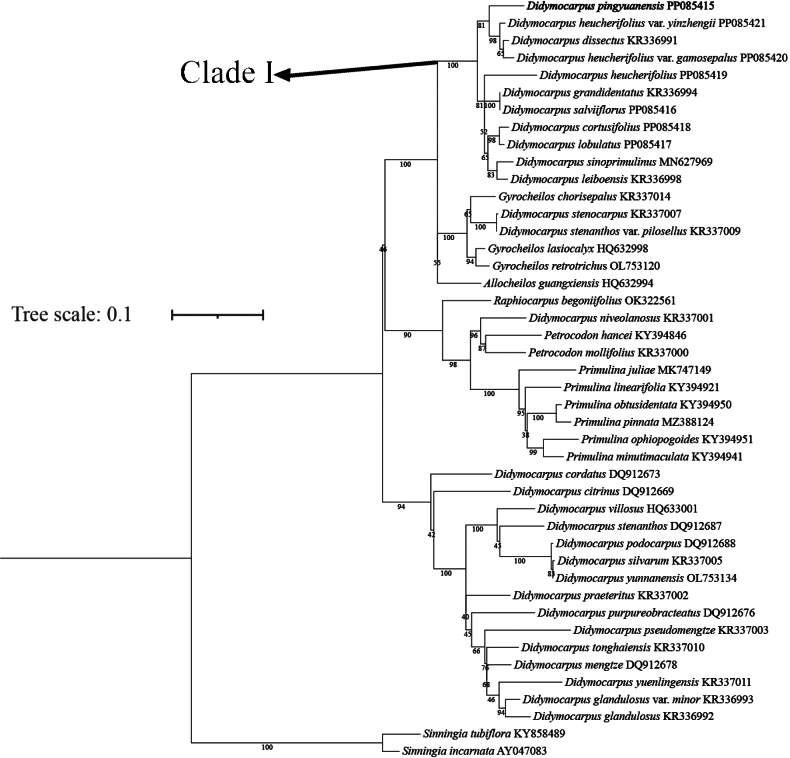
Maximum Likelihood (ML) tree based on ITS sequences of the new species *Didydmocarpuspingyuanensis* and related species. Values of bootstrap support are shown along the branches. The new species described in this study is shown in bold.

The aligned length of the ITS sequences was 730 bps. The topologies of the Bayesian and ML trees are incongruent. The BI tree is displayed below (Fig. 1), ML tree is displayed in Appendix 1: Fig. A1. The topology indicates that DidymocarpussectHeteroboea is paraphyletic, and eight taxa of DidymocarpussectHeteroboea belong to clade I (Fig. 1. PP = 1, BS = 100%). In this clade, the putative new species forms a sister group with *Didymocarpusdissectus*, D.heucherifoliusvar.gamosepalus and D.heucherifoliusvar.yinzhengii (Fig. 1. PP = 1, BS = 81%), while D.heucherifoliusvar.heucherifolius belongs to another group (Fig. 1, PP = 1, BS = 81%).

### ﻿Morphological comparison

In Didymocarpussect.Heteroboea, most species exhibit similar vegetative characteristics, except for *D.dissectus*, whose leaf margin is irregularly and distinctly 3- or 4-lobed in the distal 1/3–1/2. There are two varieties, D.heucherifoliusvar.gamosepalus and D.heucherifoliusvar.heucherifolius distributed in Guangdong Province, China. Both *D.pingyuanensis* and D.heucherifoliusvar.gamosepalus share the same distribution area, namely Pingyuan County in Guangdong, and both are distributed within the Danxia landscape. During the field investigation, we also found D.heucherifoliusvar.heucherifolius in Wuzhishi scenic spot, which is about 50 km from the locality of *D.pingyuanensis*. However, *D.pingyuanensis* can be distinguished from D.heucherifoliusvar.heucherifolius by its glabrous corolla. Furthermore, it can be distinguished from D.heucherifoliusvar.gamosepalus by its shorter corolla, and calyx deeply 5-lobed to about three quarters of the calyx length, lobes equal, obovate, apex rounded, rarely cuspidate, overlapping at margin.

Although *Didymocarpuspingyuanensis* and *D.salviiflorus* share calyx lobed and overlapping at margin, however, the former is deeply 5-lobed to about three quarters of the calyx length, contrasting with the latter 5-lobed to about half of the calyx length from the base. Additionally, *D.pingyuanensis* can also be distinguished from *D.salviiflorus* by bracts free, elliptic, and corolla glabrous outside.

**Figure 2.**
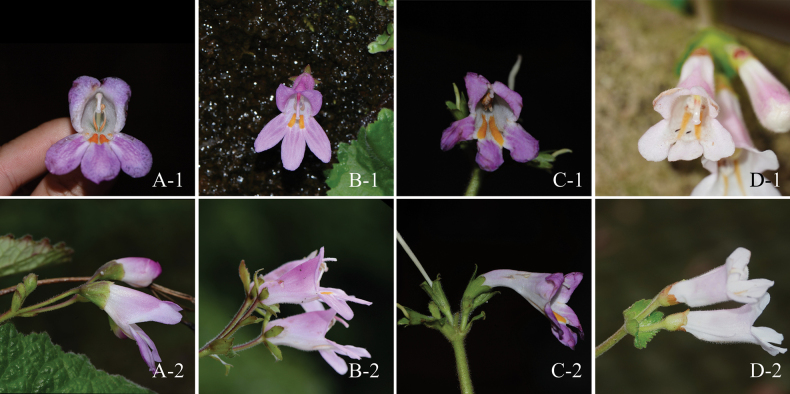
Morphological differences between *Didymocarpuspingyuanensis*, D.heucherifoliusvar.heucherifolius, D.heucherifoliusvar.gamosepalus, and *D.salviiflorus***A***D.pingyuanensis***B**D.heucherifoliusvar.heucherifolius**C**D.heucherifoliusvar.gamosepalus**D***D.salviiflorus*; **1**. front view of corolla; **2**. lateral view of corolla, showing calyx and bracts (Photographers: **A–C** by Qiang Fan **D** by Ling-Han Yang).

Differences between the putative species and its morphologically related species, Didymocarpusheucherifoliusvar.heucherifolius, D.heucherifoliusvar.gamosepalus, and *D.salviiflorus* are shown in the following identification table (Table 1) and Fig. 2.

**Table 1.** Morphological differences between the species *Didymocarpuspingyuanensis*, D.heucherifoliusvar.heucherifolius, D.heucherifoliusvar.gamosepalus, *D.salviiflorus*.

Character	*Didymocarpuspingyuanensis*	D.heucherifoliusvar.heucherifolius	D.heucherifoliusvar.gamosepalus	*D.salviiflorus*
**Shape of calyx**	deeply 5-lobed to about three quarters of the calyx length, obovate, overlapping at margin	5-lobed to the base, broadly lanceolate to oblanceolate-linear or triangular	5-lobed from middle to above middle	5-lobed to about half of the calyx length from the base, depressed oblong, overlapping at margin
**Bracts**	bracts free, elliptic, 5–12 mm, serrate, long ciliate	bracts free, elliptic, 5–10 mm, serrate, long ciliate	bracts free, 4–8 mm, serrate, long ciliate	bracts free to connate, semiorbicular, ca. 5 mm, margin sparsely crenate
**Size of corolla**	1.8–3.8 cm long	2.5–3.2 cm long	3.6–4.3 cm long	2.5–3 cm long
**Indumentum of corolla**	glabrous	puberulent	glabrous	puberulent
**Staminodes**	1.5–4 mm from the base	1–2 mm from the base	1–1.6 cm from the base	3–5 mm from the base

### ﻿Taxonomic treatment

#### ﻿ Didymocarpus pingyuanensis

Taxon classificationPlantaeLamialesGesneriaceae

Ling H.Yang, Q.Fan & F.Wen sp. nov.

93FD7193-DF47-50CD-9BA1-191E3E39F9F8

urn:lsid:ipni.org:names:77345325-1

Figs 3 , 4

##### Diagnosis.

*Didymocarpuspingyuanensis* is similar to *D.heucherifolius var. gamosepalus* and *D.salviiflorus* in having a similar zygomorphic corolla and pink to pinkish-purple funnel-shaped to tubular corolla tube, but can be distinguished from D.heucherifoliusvar.gamosepalus by its corolla size 1.8–3.8 cm long (*vs.* 3.6–4.3 cm long), calyx deeply 5-lobed to about three quarters of the calyx length, apex rounded, rarely cuspidate, overlapping at margin (*vs.* 5–lobed from middle to above middle); from D.salviiflorus, it differs by having calyx 5-lobed to about three quarters of the calyx length (*vs.* 5-lobed to about half of the calyx length from the base), bracts free, elliptic (*vs.* bracts free to connate, semiorbicular), and corolla glabrous outside (*vs.* puberulent outside).

##### Type.

China. Guangdong Province: Meizhou City, Pingyuan Town, 24°32'N, 115°50'E, 491 m a.s.l., 1 April 2023 (fl.), *Qiang Fa, Xing-yue Zhang, Li-juan Liao, Jie-hao Jin, Ling-han Yang DNPC 3352* (holotype: SYS!; isotypes: IBK! IBSC! SYS!)

##### Description.

Acaulescent perennial herb. ***Rhizome*** horizontal, 2–4 cm long, ca. 1 cm in diameter. ***Leaves*** 4–9 basal, clustered at the apex of the rhizome, whorled; leaf blades chartaceous, orbicular-ovate to triangular, 4–11 cm long, 4.5–12 cm wide, apex slightly acute, base cordate, margin lobed (lobes 18–21, irregularly triangular, with irregular teeth), upper surface densely cover with white pilose hairs, green, lower surface with sparsely short hairs and pilose brown hairs confined to the veins, pale green, basal veins 5, palmate; petiole terete, pale green, 2.5–9 cm long, densely covered with pilose brown hairs, ***Cymes*** axillary, usually 3–6 on a stem, pseudodichotomous, scapiform, each 2- to numerous flowered; Peduncle 10–18 cm long, covered with white dense short hairs and sparsely pilose brown hairs; ***Bracts*** free, elliptic, 5–12 mm long, serrate, long ciliate; Pedicel up to ca. 2.5 cm long, with short hairs; ***Calyx*** deeply 5-lobed to about three quarters of the calyx length, lobes equal, obovate, apex rounded, rarely cuspidate, overlapping at margin, 6–9 mm long, 4.5–6 mm wide at the widest part and 2–3 mm wide at the base, lobes margin denticulate, puberulent outside, glabrous inside. ***Corolla*** zygomorphic, pink to magenta, up to ca. 3.8 cm long; glabrous outside, inside with glandular puberulent hairs from the throat to the corolla base and two wide bright yellow strips at the throat; tube funnel-shaped to tubular, 1.2–2.3 cm long, ca. 6.5 mm in diameter at base, ca. 1.1 cm in diameter at throat; limb distinctly 2–lipped, adaxial lip 2-lobed to near middle, 0.6–1 × ca. 1.3 cm, obliquely triangular, abaxial lip 3-lobed to base, lobes rounded or oblong, ca. 0.9 × 1.6 cm, more or less equal. ***Stamens*** 2, adnate to corolla ca. 2 cm above the base of the corolla tube; filaments white, 8–13 mm long, slightly geniculate above base, swollen at middle, glabrous with glandules on the surface; anthers pale yellow, ca. 3 mm long, cohering face to face, white woolly. ***Staminodes*** 3, adnate to 1.5–4 mm above the base of the corolla tube, 0.4–0.8 mm long, white, glabrous. ***Pistil*** 2.2–3 cm long, puberulent; ovary white, cylindrical, puberulent; style ca. 2.3 mm long; stigma 1, cephaloid, centrally sunken, undivided, translucent. ***Capsule*** purplish-red when young, linear-cylindrical, glandular puberulent, up to ca. 9 cm long.

**Figure 3.**
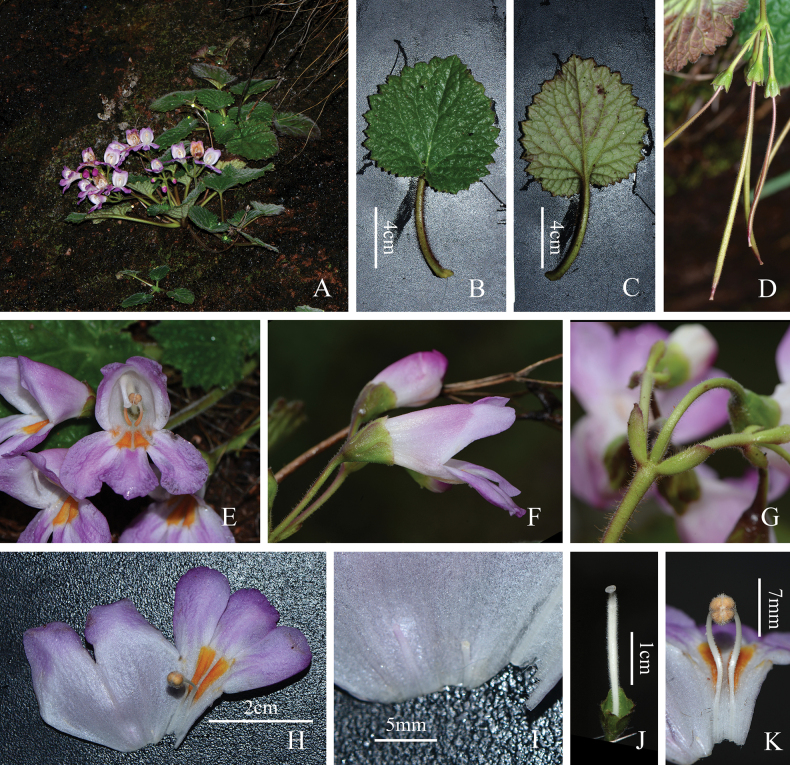
*Didymocarpuspingyuanensis***A** habitat in flowering **B** adaxial surface view of leaf blade **C** abaxial surface view of leaf blade **D** capsule **E** front view of corolla **F** lateral view of corolla, showing calyx deeply 5-lobed to about a quarter of the calyx length from the base **G** bracts **H** opened corolla **I** staminodes **J** pistil **K** stamens (Photographers: **A–K** by Qiang Fan).

##### Phenology.

The flowering of *Didymocarpuspingyuanensis* is from April to May; and the fruiting is in June.

##### Distribution and habitat.

*Didymocarpuspingyuanensis* so far has only been found on three Danxia landscapes within Pingyuan County. It is locally abundant and endemic to a narrow area near the type locality. This species prefers moist, but sunny cliffs, at an elevation of 100–500 m a.s.l. In Pingyuan, the average temperature is 21.7 °C, and the average annual precipitation is 1637 mm.

**Figure 4.**
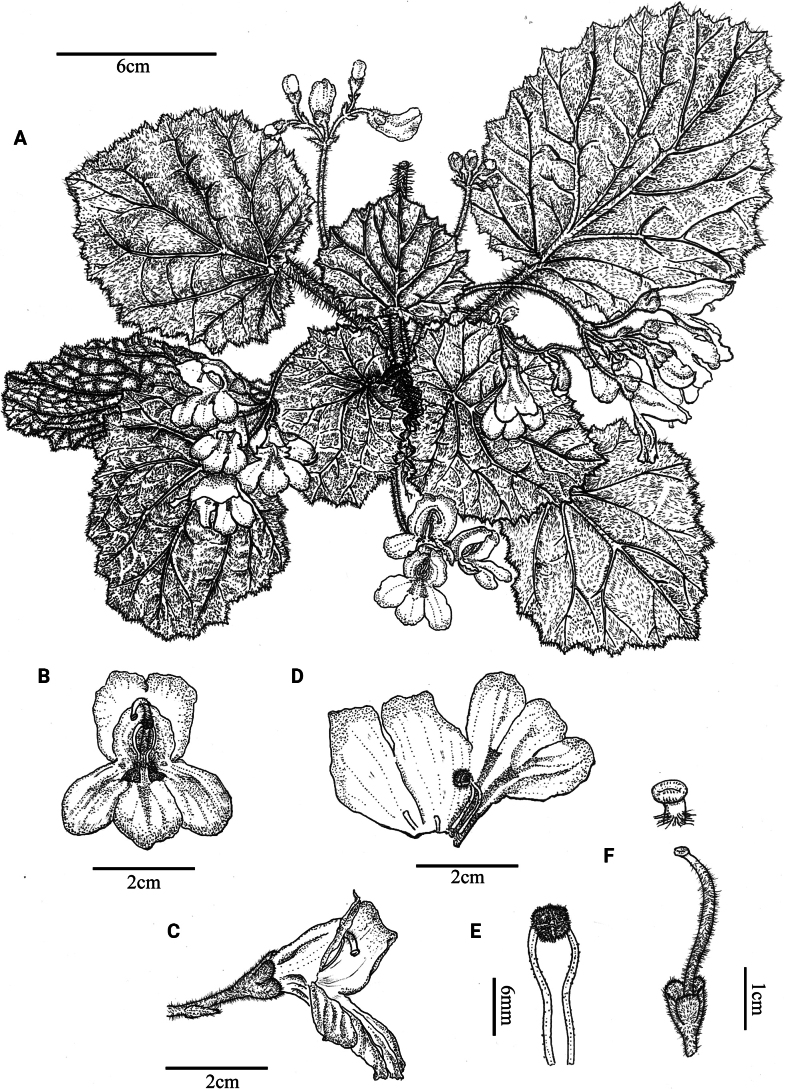
*Didymocarpuspingyuanensis* Ling H. Yang, Q. Fan & F. Wen **A** habit **B** flower in front view **C** flower in lateral view **D** opened corolla, showing stamens and staminodes **E** stamens **F** pistil and stigma (Drawn by Rong-En Wu).

##### Etymology.

The specific epithet refers to the type locality, Pingyuan County, Guangdong Province, China. The Chinese name of the new species is here given as 平远长蒴苣苔 (Píng Yuǎn Cháng Shuò Jù Tái).

##### Additional specimens examined.

*Didymocarpuspingyuanensis* (paratypes): China, Guangdong, Pingyuan: Nantai Mountain, 24°33'N, 115°53'E, 168 m a.s.l., 6 May 2023, *Qiang Fan, Zheng-Fei Li, Ling-Han Yang 20212* (SYS); Nantai Mountain, 24°32'N, 115°50'E, 491 m a.s.l., 7 May 2023, *Qiang Fan, Zheng-Fei Li, Ling-Han Yang 20219* (SYS); Dahebei scenic spot, 24°36'N, 115°49'E, 300 m a.s.l., 7 May 2023, *Qiang Fan, Zheng-Fei Li, Ling-Han Yang 20221* (SYS).

*Didymocarpusheucherifolius*: China, mountains between Shicheng in southeastern Jiangxi and Ninghua in western Fujian, 1200 m a.s.l., 7 May 1921, *Te-Hui Wang* (IBSC0005130, isotype).

*Didymocarpussalviiflorus*: China, Zhejiang, Lishui, 19 April 1930, *Guan-Guang Zhong* (00030758, isotype).

Didymocarpusheucherifoliusvar.yinzhengii: China, Hunan, Yongxing, 26°2'28″N, 113°9'8″E, 140 m a.s.l., 13 May 2023, *Qiang Fan, Zheng-Fei Li, Ling-Han Yang 20265* (SYS).

## ﻿Discussion

Morphologically, *Didymocarpuspingyuanensis* can be distinguished from other species within this genus belonging to Didymocarpussect.Heteroboea by calyx deeply 5-lobed to about a quarter of the calyx length from the base, lobes equal, obovate, apex rounded, rarely cuspidate, overlapping at margin. Initially, *Didymocarpuspingyuanensis* seems morphologically like a new variety of D.heucheifoliusvar.heucheifolius, because only D.heucheifoliusvar.heucheifolius and D.heucherifoliusvar.gamosepalus distributed in Guangdong in Didymocarpussect.Heteroboea. In addition, *Didymocarpuspingyuanensis* shares corolla glabrous outside with D.heucherifoliusvar.gamosepalus and D.heucherifoliusvar.yinzhengii, both of them were varieties of *D.heucherifolius*. However, according to the phylogenetic trees, *D.pingyuanensis*, two varieties of *D.heucherifolius* and *D.dissectus* form a sister group, while D.heucherifoliusvar.heucherifolius forms a sister group with *D.cortusifolius*, *D.salviiflorus*, *D.lobulatus*, *D.grandidentatus*, *D.leiboensis* and *D.sinoprimulinus*. Thus, we suppose *D.pingyuanensis* is a distinct species (Fig. 1), and elevate the two varieties of *D.heucherifolius* to species level, namely *D.yinzhengii* and *D.gamosepalus*.

### ﻿New combination

#### ﻿ Didymocarpus yinzhengii

Taxon classificationPlantaeLamialesGesneriaceae

(J.M.Li & S.J.Li.) Ling H.Yang, Q.Fan & F.Wen comb. nov.

6BACB540-8112-5C64-A9CC-7A77A0451619

urn:lsid:ipni.org:names:77345326-1

≡ Didymocarpusheucherifoliusvar.yinzhengii J.M.Li & S.J.Li. Phytotaxa 156 (3): 187. 2014.

##### Type.

China. Hunan: near Yongxing County. alt. 300 m, 26°17'10"N, 113°11'25"E, 6 May 2011, *Jia-Mei Li 1105062* (holotype: HEAC!); ibid. *Jia-Mei Li 11501* (paratype: IBK!).

#### ﻿ Didymocarpus gamosepalus

Taxon classificationPlantaeLamialesGesneriaceae

(Xin Hong & F.Wen) Ling H.Yang, Q.Fan & F.Wen comb. nov

D13750EE-F083-59DC-9D4A-2A4629D55DED

urn:lsid:ipni.org:names:77345327-1

≡ Didymocarpusheucherifoliusvar.gamosepalus Xin Hong & F.Wen. PhytoKeys 128: 34. 2019.

##### Type.

China. Guangxi Province, cultivated in the nursery of Gesneriad Conservation Center of China (GCCC), introduced from north of Guangdong Province: Pingyuan County, Meizhou City, growing in rocky crevices at the foot of a calcareous sedimentary rocky hill. 22 February 2019, flowering, *WF20190222-05* (holotype: IBK!; isotype: AHU!)

## Supplementary Material

XML Treatment for Didymocarpus pingyuanensis

XML Treatment for Didymocarpus yinzhengii

XML Treatment for Didymocarpus gamosepalus

## ﻿Additional information

### Author ORCIDs

Ling-Han Yang https://orcid.org/0009-0008-8895-1543

Fang Wen https://orcid.org/0000-0002-3889-8835

Qiang Fan https://orcid.org/0000-0003-4254-6936

### Data availability

The newly obtained sequences of *Didymocarpuscortusifolius*, *D.salviiflorus*, *D.lobulatus*, D.heucherifoliusvar.heucherifoliu, D.heucherifoliusvar.yinzhengii, D.heucherifoliusvar.gamosepalus have been submitted to the NCBI website. The ITS sequence data supporting the findings of this study are available in Fig. 1.

## References

[B1] Doyle JJ Doyle JL (1987) A rapid DNA isolation procedure for small quantities of fresh leaf tissue. Phytochemical Bulletin 19(1): 11–15. http://worldveg.tind.io/record/33886/files/d015081.pdf

[B2] Lee SY Xu KW Huang CY Lee JH Liao WB Zhang YH Fan Q (2022) Molecular phylogenetic analyses based on the complete plastid genomes and nuclear sequences reveal *Daphne* (Thymelaeaceae) to be non-monophyletic as current circumscription. Plant Diversity 44(3): 279–289. 10.1016/j.pld.2021.11.001 35769588 PMC9209861

[B3] Li JM Li SJ (2014) Didymocarpusheucherifoliusvar.yinzhengii (Gesneriaceae), a new taxon from Hunan, China. Phytotaxa 156(3): 187–190. 10.11646/phytotaxa.156.3.10

[B4] Li ZL Huang Z Chen DW Hong X Wen F (2023) A new combination and a new synonym of Gesneriaceae in China. PhytoKeys 232: 99–107. 10.3897/phytokeys.232.108644 37746323 PMC10517411

[B5] Möller M Clark JL (2013) The State of Molecular Studies in the Family Gesneriaceae: A Review. Selbyana 31: 95–125. https://www.jstor.org/stable/24894284

[B6] Ronquist F Teslenko M van der Mark P Ayres DL Darling A Höhna S Larget B Liu L Suchard MA Huelsenbeck JP (2012) MrBayes 3.2: Efficient Bayesian phylogenetic inference and model choice across a large model space. Systematic Biology 61(3): 539–542. 10.1093/sysbio/sys029 22357727 PMC3329765

[B7] Tamura K Stecher G Kumar S (2021) MEGA11: Molecular Evolutionary Genetics Analysis Version 11. Molecular Biology and Evolution 38(7): 3022–3027. 10.1093/molbev/msab120 33892491 PMC8233496

[B8] Wang WT Pan KY Li ZY (1990) Gesneriaceae. In: Wang WT (Ed.) Flora Reipublicae Popularis Sinicae, vol.69. Science Press, Beijing, 420–451.

[B9] Weber A Brutt BL (1998) Remodelling of *Didymocarpus* and Associated Genera (Gesneriaceae). Beitrage zur Biologie der Pflanzen 70: 293–363.

[B10] Weber A Burtt BL Vitek E (2000) Materials for a revision of *Didymocarpus* (Gesneriaceae). Annalen des Naturhistorischen Museums in Wien. Serie B, Fur Botanik und Zoologie 102: 441–475.

[B11] Weber A Wei YG Puglisi C Wen F Mayer V Möller M (2011) A new definition of the genus *Petrocodon* (Gesneriaceae). Phytotaxa 23(1): 49. 10.11646/phytotaxa.23.1.3

[B12] Wen F Qiu YL Huang J Zhao B Wei YG (2013) *Didymocarpusdissectus* sp. nov. (Gesneriaceae) from Fujian, eastern China. Nordic Journal of Botany 31(3): 316–320. 10.1111/j.1756-1051.2012.00057.x

[B13] White TJ Bruns T Lee S Taylor J Innis M Gelfand D Sninsky J (1990) Amplification and Direct Sequencing of Fungal Ribosomal RNA Genes for Phylogenetics. In: Pcr Protocols: a Guide to Methods and Applications, vol. 18. Academic Press, New York, 315–322. 10.1016/B978-0-12-372180-8.50042-1

[B14] Xie WY Zhou JJ Hong X Wen F (2020) *Didymocarpuslobulatus* (Gesneriaceae), a new species from Zhejiang Province, East China. PhytoKeys 157: 145–153. 10.3897/phytokeys.157.30349 32943977 PMC7471605

[B15] Xu WJ Qin WH Wang ZQ Li ZL Fu LF Hong X (2019) A new variety of *Didymocarpus* (Gesneriaceae) from Guangdong, China. PhytoKeys 128: 33–38. 10.3897/phytokeys.128.35446 31388327 PMC6675924

